# Targeting androgen receptor signaling with MicroRNAs and Curcumin: a promising therapeutic approach for Prostate Cancer Prevention and intervention

**DOI:** 10.1186/s12935-021-01777-3

**Published:** 2021-01-26

**Authors:** Zeeshan Javed, Khushbukhat Khan, Amna Rasheed, Haleema Sadia, Muhammad Naeem Shahwani, Asma Irshad, Shahid Raza, Bahare Salehi, Javad Sharifi-Rad, Hafiz A. R. Suleria, Natália Cruz-Martins, Cristina Quispe

**Affiliations:** 1 Office for Research Innovation and Commercialization, Lahore Garrison University, DHA, Sector-C, Phase VI, Lahore, Pakistan; 2 Atta-ur-Rahman School of Applied Biosciences (ASAB), National University of Sciences and Technology (NUST), 44000 Islamabad, Pakistan; 3 School of Basic Medical Sciences, Lanzhou University, 730000 Lanzhou, PR China; 4 Department of Biotechnology, Balochistan University of Information Technology, Engineering and Management Sciences, Quetta, Pakistan; 5 Department of Life Sciences, University of Management Sciences, Lahore, Pakistan; 6 Medical Ethics and Law Research Center, Shahid Beheshti University of Medical Sciences, Tehran, Iran; 7 Phytochemistry Research Center, Shahid Beheshti University of Medical Sciences, Tehran, Iran; 8 Facultad de Medicina, Universidad del Azuay, Cuenca, Ecuador; 9 School of Agriculture and Food, Faculty of Veterinary and Agricultural Sciences, The University of Melbourne, 3010 Parkville, VIC Australia; 10 Faculty of Medicine, University of Porto, Alameda Prof. Hernâni Monteiro, 4200-319 Porto, Portugal; 11 Institute for Research and Innovation in Health (i3S), University of Porto, 4200-135 Porto, Portugal; 12 Laboratory of Neuropsychophysiology, Faculty of Psychology and Education Sciences, University of Porto, 4200-135 Porto, Portugal; 13 Facultad de Ciencias de la Salud, Universidad Arturo Prat, Avda. Arturo Prat 2120, 1110939 Iquique, Chile

**Keywords:** MicroRNAs, Curcumin, Nanoformulations, Diagnostic markers, Androgen receptor signaling, Prostate cancer

## Abstract

Prostate cancer (PC) is a multifactorial disease characterized by the abrogation of androgen receptor signaling. Advancement in microbiology techniques has highlighted the significant role of microRNAs (miRNAs) in the progression of PC cells from an androgen-dependent to an androgen-independent state. At that stage, prostate tumors also fail to respond to currently practiced hormone therapies. So, studies in recent decades are focused on investigating the anti-tumor effects of natural compounds in PC. Curcumin is widely recognized and now of huge prestige for its anti-proliferative abilities in different types of cancer. However, its limited solubility, compatibility, and instability in the aqueous phase are major hurdles when administering. Nanoformulations have proven to be an excellent drug delivery system for various drugs and can be used as potential delivery platforms for curcumin in PC. In this review, a shed light is given on the miRNAs-mediated regulation of androgen receptor (AR) signaling and miRNA-curcumin interplay in PC, as well as on curcumin-based nanoformulations that can be used as possible therapeutic solutions for PC.

## Introduction

Prostate cancer (PC) is a leading cause of death in the male population worldwide [1]. Briefly, it is a complex disease characterized by an altered cell signaling pathway that triggers uncontrolled growth and differentiation. Different molecular cascades are crucial for normal cell growth and cell-to-cell communication [2] so that changes in such signaling pathways can trigger tumor heterogeneity and aggressiveness [3]. PC is ranked fifth among the causes of death in males, affecting the age group between 60 and 65 years [4]. The underlying causes of PC involve abrogation of androgen receptor signaling, a pivotal player in regulating and maintaining the normal growth of the prostate gland [5]. Radiation therapy and surgery are the two currently available therapeutic options for PCs and androgen deprivation therapy (ADT) for persistent advanced metastatic disease cases. Despite the ADT and other therapeutic options available, tumor recurrence is common and can lead to castration-resistant (CRPC) [6]. Androgen receptor (AR) is an important mediator of prostate gland growth and development, which is vital for prostate carcinogenesis and PC progression [7]. AR is predominantly expressed in all PC cases [7]. Several studies have shed light on the relationship between the cell levels of AR, PC metastasis, and progression. Aberrant AR signaling has been considered a pivotal player in transforming clinically localized hormones into aggressive-resistant cancers [8]. Prostate-specific antigen (PSA) is a diagnostic marker currently employed to measure the actual contortion caused by the abrogated AR signaling pathway [9]. A plethora of studies have delineated the mechanisms responsible for therapeutic failure in cases of aggressive PC [10, 11]. Mutations in AR receptor, AR overexpression, intracrine production, contorted expression of enzymes and cofactors of AR, autonomous activation of AR signaling by cytokines in the absence of androgen ligands, and the presence of multiple splice variants of AR are the main causative agents responsible for the therapeutic failure [12, 13]. Thus, AR signaling abrasions are the main driving factors behind PC progression and drug resistance [13].

Several therapeutic strategies are analyzed in preclinical studies to find an appropriate and effective drug resistance and tumor recurrence solution. Treatment with glutamate inhibitors was studied in xenograft mice, and findings of the study depicted the restoration of radiosensitivity of grafted tumor cells [14]. Likewise, combine the administration of chemotherapy drugs is another strategy that is being evaluated *in vitro.* Oxaliplatin, patulin, and emetine reported having a synergistic effect on the viability of tumor cells, which depended on the amount and sequence of administered drugs [15]. Recent studies have also reported the influence of miRNAs on the molecular landscape of tumor cells. They are important transcription, translation, transportation, and ubiquitination mediators [16]. It has come to light less lately that miRNAs can influence AR signaling at various levels. Their interplay with AR signaling can be valuable for devising new diagnostic and therapeutic strategies for PC [17].

The modulatory influence of several natural compounds on miRNA functioning in cancers is demonstrated in numerous investigations. Similarly, by enhancing the activity of tumor suppressor miRNAs or by silencing the expression of oncomiRNAs, natural compounds regulate the signal transduction through many cellular pathways. The extracts of *Pygeum africanum* are reported to have antagonistic activity against the androgen receptor in benign prostate lesions [18]. Co-administration of ursolic acid, resveratrol, and curcumin in prostate xenograft mice led to a reduction in tumor size by modulating mTOR and glutamine pathway [19]. Similarly, bitter melon extracts also regulate the mTOR pathway to induce autophagy, ultimately resulting in cell death. Extracts of bitter melon also induce modulation of natural killer cells and Treg cells to inhibit proliferation of tumor cells [20, 21]. Curcumin is a natural compound derived from the roots of the plant *Curcuma longa* L. [22]. Indeed, the curcumin mediated modulation of miRNAs for progressive control of cancer growth and proliferation is an exciting avenue. However, a few studies have evidenced the involvement of curcumin in the modulation of miRNAs in different cancers [23–25].

Nonetheless, curcumin has been reported to have tremendous anti-proliferative capabilities [26]. Despite its hydrophobic nature, limited bioavailability and rapid metabolism are viewed as stumbling blocks that have hampered its therapeutic activity [26]. In such a way, nanoformulations have been considered to improve curcumin delivery at target sites [27]. Nanodelivery systems have been extensively used in the modern pharmaceutical industry because of their limited cytotoxicity and high specificity; so, curcumin nanoformulations can be effectively used to treat PC and other diseases [28]. In this sense, this review aims to provide an overview of the AR signaling in PC, its interaction with miRNAs and curcumin, and the currently available curcumin nanoformulations that can be implemented as possible therapeutic solutions in both normal and aggressive forms of PC.

### Androgen receptor (AR) signaling: an overview

**Fig. 1:**
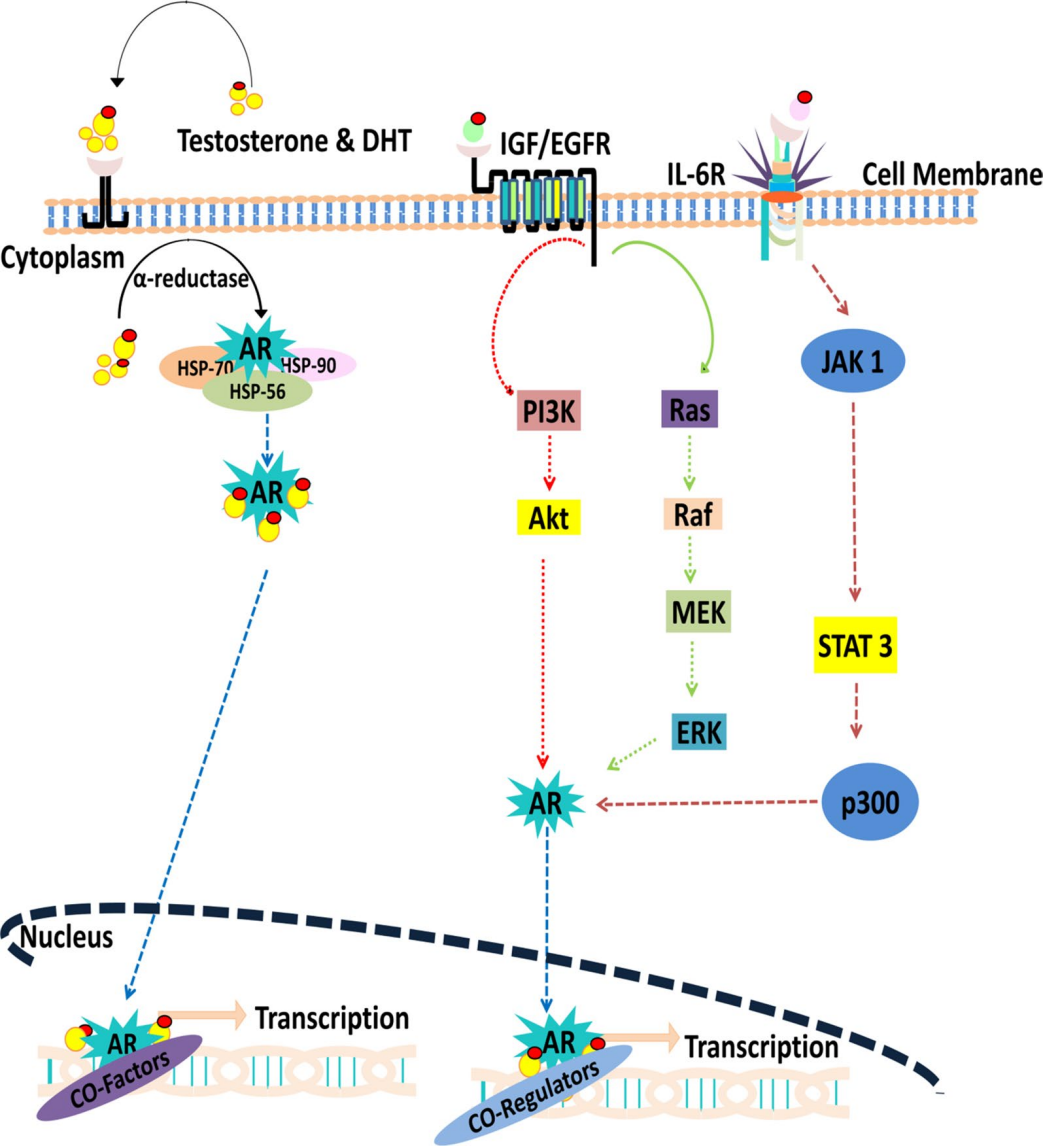
A description of both ligand dependent- and independent-AR signaling. Binding of DHT to androgen facilitates its transportation to the nucleus, where it exerts its influence through regulating the transcription of key genes with the help of cofactors and coregulators. In ligand AR-independent activation, AR receptor is activated by either insulin growth factor/epidermal growth factor receptor or by the interleukin-6 signaling which through various cross-talks trigger the expression of desired genes

Testosterone is a 19-carbon steroid produced in male testes with the help of adrenal glands. It belongs to the androgenic steroids, which are key modulators of several developmental and physiological responses. Testosterone is converted to dihydrotestosterone (DHT) by the action of 5α-reductase, an enzyme generated by the cytochrome P450 [29]. The DHT has been reported to be highly expressed in genital tissues and prostate glands. Together, DHT and testosterone can activate the AR signaling. However, DHT has a greater binding affinity for AR and can trigger its activation even at minimal concentrations compared to testosterone. AR is usually located in the cytoplasm when not activated by the ligand [30]. AR has been investigated to be associated with heat shock proteins (HSPs) in the cytoplasm, namely HSP-90, HSP-70, HSP-56, and other molecular chaperones [31]. This association enables both AR activation and translocation to the nucleus and cytoplasm. HSPs interact with cytoskeletal proteins, such as filamin A (FlnA) that guides AR either toward the nucleus or cytoplasm by interacting with the hinge-region of AR [31]. Androgens further enhance this interaction between AR and FlnA, and the co-localization of FlnA and AR in the presence of androgens recruits integrin-β1 [32, 33]. This recruitment promotes the activation of focal adhesion kinase (FAK) and Ras-related C3 botulinum toxin substrate 1 (RAC1). These two molecules strictly modulate cell migration in PC [34]. Research has shed light on the fact that these small molecular interactions between AR, FlnA, Rac1, and FAK trigger cell migration, driving force in PC progression and metastasis [33]. Indeed, the binding of androgens (ligands) brings conformational changes in AR, resulting in a pocket that generates an AF-2 binding surface that aid in the recruitment of coactivators and nuclear transportation of AR [33]. Once AR reaches the nuclear environment, it interacts with the AR response elements (AREs) that recruits it to the promoter region of the gene. Then, the AR transcriptional complex is completed by the addition of co-regulators that can either promote gene activation or inhibition [35]. A brief scheme of such a mechanism is provided in Fig. 1.

## MiRNAs and androgen signaling

MicroRNAs (miRNAs) are a class of small (18–22 nt), non-coding, and indigenous RNA molecules that actively regulate the expression of other genes by binding to complementary sequences in 3’UTR of mRNAs to inhibit translation. More than 2000 miRNAs, and imaginably there are a huge number of target mRNAs since each miRNA can bind multiple molecules [36]. Thus, the role of miRNAs in almost all cell processes is extensive and is being unraveled with each passing day. Briefly, the miRNAs expression is induced by a number of regulators, including androgen, ultimately used to control several physiological processes, such as apoptosis, cell division, and cancer growth and development [37].

Several AR variants (ARVs) result from alternative splicing of premature AR and are the real drive behind tumor progression. In most ARVs, the ligand-binding domain is missed; however, due to the N-terminal domain, they retain the tumor progression abilities [38, 39]. In line with the general role and mode of action, several miRNAs directly affect or are affected by androgen and androgen receptor (AR) to play a key role in various tumor regulation (Table 1). miRNAs, along with androgen signaling, can work as both tumor suppressors and promoters. For example, androgen-dependent overexpression of miR-125b is linked to PC progression. However, the transient expression of miR-125b can induce androgen-independent PC by inhibiting the pro-apoptotic Bak1 [40]. A recent study has shown that curcumin-encapsulated polymersome nanoparticles (CPNs) can deregulate miR-125b and play a role in suppressing breast cancer (BC) [41]. Also, androgen-induced release and binding of AR can directly bind to the promoter region miR-21, a well-known oncomiR miRNA [42], which upregulates its expression, resulting in CAP progression [43]. AR-induced upregulation of miR-21 results in deregulation of tumor suppressor Pdcd4, since miR-21, targets its 3’UTR in PC progression [44]. Curcumin inhibits miR-21 expression via AP-1, resulting in tumor suppressor Pdcd4 stabilization in colorectal cancer [45]. Another study reported at least 10 androgen-responsive miRNAs, including miR-141, miR-200a, and miR-148a, that promote PC. Among these miRNAs, miR-148a binds to the 3′-untranslated region of cullin-associated and neddylation-dissociated 1 (CAND1) mRNA. It inhibits the CAND1 expression, a negative regulator of SKP1-Cullin1-F-box (SCF) ubiquitin ligases, by binding to the 3′-untranslated region of CAND1 mRNA [46]. Contrarily, miR-148a has been linked to abrogation of epithelial-mesenchymal transition (EMT), which plays a critical role in cancer invasion and metastasis, at the same time that it exerts a suppressive role in pancreatic cancer cells invasion by targeting Wnt10b and inhibiting the Wnt/β-catenin signaling pathway [47]. Thus, the role of miR-148a varies in the progression of different kinds of cancers. Interestingly, just like its role in different kinds of cancer progression, miR-148a expression is differently regulated by various curcuminoids [48]. More specifically, curcumin I (diferuloylmethane) does not affect the miR-148a expression, while curcumin II (desmethoxycurcumin) downregulates, while curcumin III (bisdemethoxycurcumin) upregulates the miR-148a expression [48]. Similarly, another study has unraveled the role of several miRNAs, including miR-19a, miR-27a, and miR-133b, in PC progression in LNCaP cells in response to androgen. It was further unveiled that all these miRNAs have different mechanisms on cancer progression. For instance, miR-19a promotes PC by inhibiting a number of proteins, including SUZ12, RAB13, SC4MOL, PSA, P, and ABCA1, whereas miR-27a does the same by inhibiting ABCA1 and PDS5B. Lastly, miR-133b inhibits CDC2L5, PTPRK, RB1CC,1, and CPNE3 in its course to promote PC [49]. Similarly, miR-30 directly inhibits the AR expression and miR-30, enhancing the AR expression and androgen-independent cell growth, ultimately acting as a tumor suppressor in PC [50]. Among these miRNAs, the effect of curcumin has been studied in miR-27, and it was found that curcumin suppresses the miR-27a in colorectal cancer [51, 52]. Interestingly, some miRNAs are also able to regulate androgen signaling. In this regard, a comprehensive proteomic profile revealed at least 12 miRNAs able to regulate AR expression, playing a role in cancer progression [53]. This study further reported that miR-135a directly binds to AR and causes its inhibition that could be restored by androgen depleted conditions. Similarly, miR-34 expressions are negatively correlated with AR expression, indicating a suppressive role of miR-34 [54]. A study has also shown that curcumin can increase the miR-34a expression in SGC-7901 cells and inhibit cell proliferation, migration, and invasion. Curcumin could also significantly inhibit cell cycle progression in G0/G1-S phase and increase the number of cells in the G0/G1 phase, downregulating the Bcl-2, CDK4, and cyclin D1 protein expression in cells and tissues [55]. However, the regulatory role of curcumin in the aforementioned cell processes and their link with the miR-34 family remains elusive.

**Table 1:** List of miRNAs regulating AR signaling in PC

MiRNAs	Expression	Comment	References
miR-125b	Up regulated	Androgen independent growth	[40]
miR-21	Up regulated	Expression is promoted by AR signaling	[43]
miR-30	Down regulated	Inhibit AR	[50]
miR-34	Down regulated	Inhibit AR	[54]
miR-135a	Down regulated	Inhibit AR	[53]
miR-205a	Down regulated	Inhibit AR	[56]

Another miRNA, named miR-205, is also being studied, and evidence highlight that it negatively correlates with AR expression and deregulation in PC [56]. In such a way, it was stated that poly(lactic-co-glycolic acid)-curcumin nanoparticles dose can significantly induce the miR-205 expression in CaPat at the same time that inhibits nuclear β-catenin and AR expression, indicating the therapeutic significance of curcumin [57]. Another study has reported that curcumin intake could significantly upregulate the expression of the miR-205 family, specially mmu-miR-205-5p, i.e., 100 times higher than controls [58].

Besides PC, androgen signaling is also involved in the progression of other tumors. For instance, miR-363 is involved in BC regulation through AR induction in a feedback loop-mediated activation of the IQWD1 gene [59]. Similarly, miR-100 and miR-125 expression is negatively correlated with AR in BC progression. Indeed, the downregulation of both miRNAs are linked to the extracellular release of metalloprotease-13 (MMP13), which is inhibited by transient expression of miR-100 and miR-125, also reversing the BC progression by AR [60]. AR negatively induces lncRNA in the progression of triple-negative breast cancer (TNBC). AR negative induction of lncRNA (ARNILA) promotes epithelial-mesenchymal transition (EMT) and works as competent for miR-204 to facilitate the expression of its target gene Sox4, an EMT inducer [61].

**Fig. 2:**
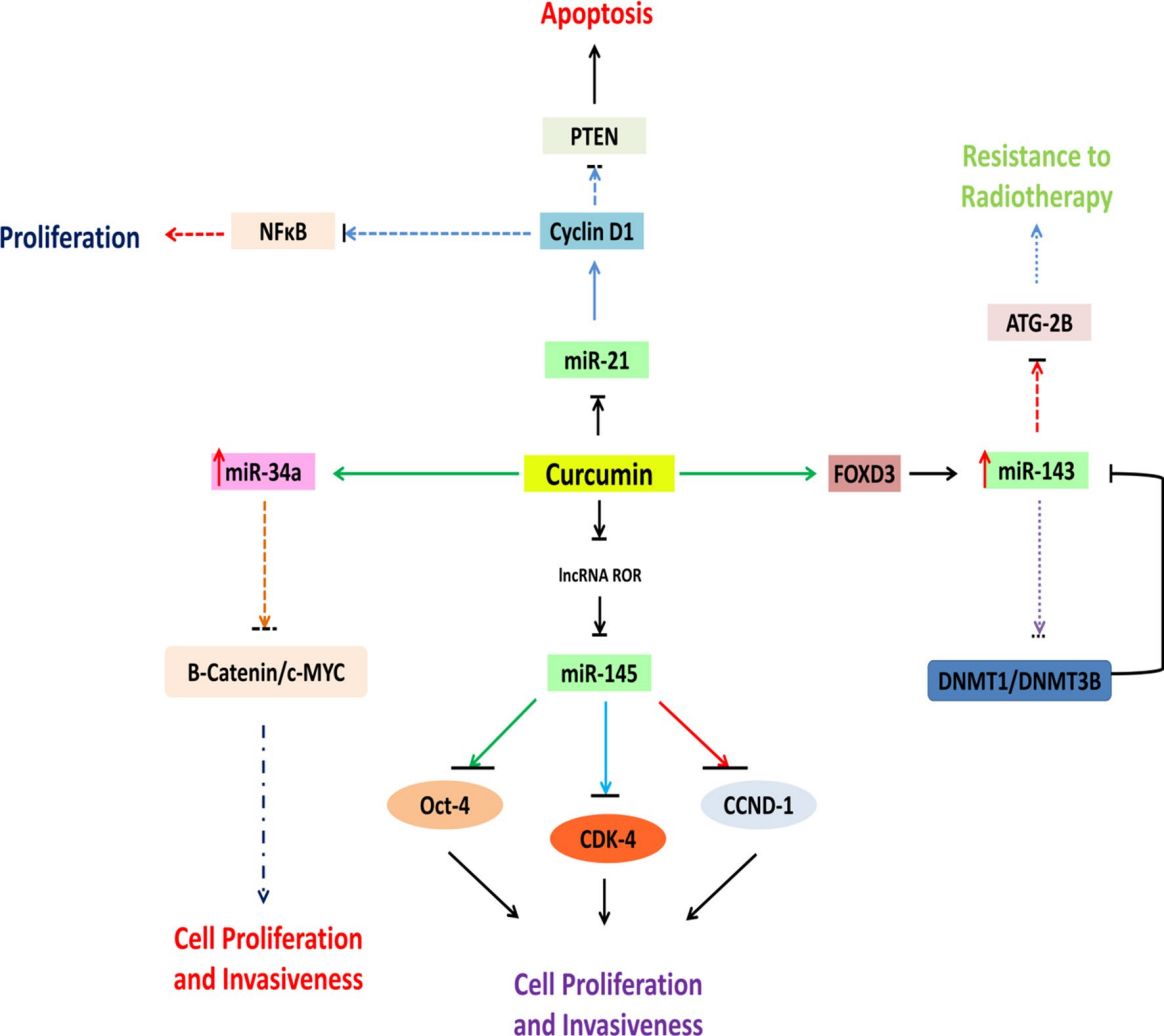
Curcumin mediates miRNA regulation in PC. Curcumin treatment modulates several PC pathways by up- or down-regulating the miRNAs associated with several cell pathways. It induces miR-143 expression via FOXD3. MiR-143 inhibits its epigenetic silencer DNMT1 and DNMT2B and also ATG2B and promotes sensitization to radiotherapy. Curcumin also promotes cell cycle arrest by inhibiting B-catenin/cmyc complex via miR-34, sequesters lncRNA-ROR and enhances miR-145 bioavailable, leading to anti-proliferation and invasiveness. Curcumin also inhibits miR-21 and induce cell proliferation suppression by blocking NF-kB pathway and apoptosis via PTEN upregulation

Interestingly. Curcumin can affect the androgen-induced miRNAs to play its role in cancer suppression. For example, curcumin can increase the miR-98 expression, which targets LIN28A, MMP2, and MMP9 and suppresses lung cancers in A549 cancer cell lines [62]. LIN28A can activate AR via c-mycregulation and promotes malignancy of ER-/Her2 + BC [63], thus providing strong evidence that curcumin can directly or indirectly interact with androgen signaling pathways and can affect tumor progression by regulating androgen signaling. Moreover, curcumin can regulate a number of miRNAs involved in androgen signaling to suppress a wide range of cancers. So, further strategies can be designed to target these miRNAs by curcumin to suppress several cancers. The above-referred mechanisms are briefly pictured in Fig. 2.

## Curcumin and miRNA interplay in PC

Various studies have revealed that the treatment with natural compounds modulates miRNA expression to promote anticancer action [64, 65]. Similarly, curcumin has also been documented to employ miRNA in preventing cancers [66]. In PC, it targets miRNA modulating different cell signaling pathways and affects cell survival, cell cycle progression, cell proliferation and death, resistance to therapy, metastasis, and autophagy [67]. Table 2 enlists miRNAs, whose expression is altered in curcumin-treated PC cells.

**Table 2:** Curcumin regulated miRNAs and their effect on prostate cancer

Sr No	MicroRNAs	Expression	Anti-tumorigenic influence	Refs.
1	miR-34a	Increase	Cell cycle arrest	[70]
2	miR-143	Increase	Halted cell proliferation, suppression of cell invasion, re-sensitization to radiotherapy and inhibition of autophagy	[71, 72]
3	miR-770-5p miR-1247	Increase	Reduced cell migration	[74]
4	miR-145	Increase	Cell cycle arrest	[79]

In initial studies, the advantages of microarray were explored to get the expression profile of miRNAs in PC BxPC3 cell line after curcumin treatment. The expression of 29 miRNAs was reported to be dysregulated after 72 h of curcumin exposure (10 µmol/L). After validation by TaqMan real-time protein chain reaction (PCR), the expression of miR-199a* and miR-22 was found to be down-regulated and up-regulated, respectively. The study also reported that the miR-22 inhibition via the use of its anti-sense antagonist enhanced the expression of estrogen receptor 1 and SP1 transcription factor [68]. However, the anti-PC effect following up-regulation of such factors was not demonstrated in the study. But, Pasqualini et al. [69] reported Mcl-1 and LAMC1 as direct targets of miR-22 in PC, suggesting the tumor-suppressive role of this miRNA, where its restored concentration inhibited tumor invasiveness and proliferation. The authors also stated that miR-22 expression is down-regulated in AR-dependent PC [69]. So, it can be speculated that curcumin treatment can also be applied against hormone-dependent PC type.

Few studies have been conducted providing insights on the mechanism behind curcumin/miRNA interaction in PC. According to Zhu and colleagues, curcumin inhibits β-catenin and c-myc axis by up-regulating the expression of tumor-suppressor miR-34a. Curcumin-induced miR-34a expression has also been correlated with cell cycle regulation-associated proteins, such as p21, PCNA, and cyclinD1 [70]. The study outcomes suggested that curcumin-mediated miR-34a modulation induces cell cycle progression arrest. Further, the miR-143 expression has also been reported to be increased in curcumin-treated DU145, LNCaP, and PC3 cells, leading to reduced migration and growth potential and increased sensitivity to radiation therapy [71, 72]. Mechanistically, curcumin promotes miR-143 expression by inhibiting DNMT1 and DNMT3B expression and inducing hype-methylation of the miR-143 promoter [71]. A similar action of curcumin was already reported in bladder cancer, wherein *vitro* treatment led to hypomethylation of the under-expressed miR-203 promoter region and expression’ restoration[73]. Curcumin is also able to up-regulate the expression of FOXD3 in PC cell lines. Being FOXD3 a transcription factor, it interacts with the miR-143 promoter region and activates its expression [72]. In turn, elevated miR-143 expression induces post-transcriptional repression of oncogenic PGK1 and ATG2B expression, leading to tumor cells’ restricted growth and autophagy inhibition [71, 72].

Curcumin can also facilitate anti-proliferation and reduce the migration property of PC stem cells by modulating miRNAs. It brought on *in vitro* inhibition of human PC stem cells (HuPaCS cells) carcinogenicity by up-regulating miR-770-5p, and miR-1247 transcription, which is part of the *DLK1*-*DIO3* imprinted gene cluster [74]. Curcumin treatment also induces miR-34a expression [70]. In PC stem cells, the ectopic expression of miR-34a targets a population of CD44^+^PC cel, ls which prevent cancer metastasis and regeneration [75], despite the direct relation of curcumin-induced miR-34a expression and PC stem cell repression is not validated. Curcumin in other cancer stem cells is reported to modulate several signaling pathways to curb their growth. For instance, it suppressed cancer stemcells growth by targeting the hedgehog signaling cascade in bladder cancer, resulting in their death [76]. In colon cancer, curcumin treatment lowered the resistance to irinotecan therapy by act activating the intrinsic cell death pathway and promoting apoptosis of cancer stem cells [77]. Curcumin treatment-mediated miRNA regulation has also been explored in other cancers, such as in oral cancer stem cells, where minute concentrations effectively brought on stem cell proliferation inhibition by repressing expression of onco-miR-21 [78]. So, elucidating the curcumin/miRNA interplay will enhance the understanding of cancer stemness’s molecular pathology, which could be a new arena in treating several types of cancer, including PC.

On the other side, curcumin treatment has also been demonstrated to act in improving the bioavailability of miRNAs. Mechanistically, it acts on ceRNAs of regulated-miRNAs sponging them. For instance, LncRNA-ROR, being a molecular sponge of miR-145, prevents binding of Oct4 and miR-145 and promotes tumorigenicity. Curcumin, by down-regulating the lncRNA-ROR expression, improves miR-145 availability in CD44+/CD133 + HuPaCS cells, which allows Oct4 inhibition. Moreover, an elevated concentration of MiR-145 also attenuates cell cycle Cdk4 and Ccnd1 protein expression [79]. Curcumin also induces epigenetic activation of miR-145, leading to its enhanced transcription [71]. Thus, curcumin treatment in HuPaCS cells reduces cancer cells’ invasiveness and proliferation abilities.

Concomitantly, different curcumin analogs have also been addressed for their pro-apoptotic properties in PC. For example, the analog EF24 blocks signal transduction by acting on the NF-κB pathway and induce death in DU-145 cells by inhibiting oncogenic miR-21 expression. MiR-21 suppression also inhibits cyclinD1 and Ki67, leading to cell cycle arrest and enhancing the concentration of its target genes, such as PDCD4 and PTEN, which leads to cell activation apoptosis pathway [80]. Pyridine-analogs of curcumin also targets the NF-κB pathway in PC3 cells and bring on cell apoptosis at a minimum of 1 µM dose [81]. In EF24-treated DU-145 cells, up-regulation of anti-carcinogenic miRNAs miR-206, miR-10a, miR-345, and miR-409 has also been reported [80], but the exact molecular consequence of their up-regulated expression is not yet explained.

Few studies have been conducted in describing the curcumin role in modulating miRNAs expression. However, the limited data available has very much delineated the curcumin contribution in inhibiting PC cells’ growth, invasiveness, and autophagy and in restoring their sensitivity to radiation therapy. Yet, there is scope for further studies to analyze the curcumin and miRNAs interplay in halting angiogenesis in PC. Autophagy and apoptosis go side by side, so studies investigating the crosstalk of such pathways concerning the role of miRNA can promote the current understanding of molecular mechanisms in PC.

## Curcumin clinical trials

The therapeutic role of curcumin is known to mankind since the 18th century. In 1937, the first study was published that reported its use in treating human disease. The author treated 67 patients with biliary diseases by oral administration of curcunat. Although only one patient was cured entirely, side effects were reported in the rest of the patients [82]. Several clinical studies on different human diseases established their tolerability, safety, and toxicity [83, 84].

Further studies demonstrated its effectiveness alone and in combination with other clinical drugs or natural compounds in several diseases, including cancers. Phase I/II clinical trial on pancreatic cancer reported that curcumin (dose: 8 g/day) induced sensitization for gemcitabine, and combined treatment of curcumin and gemcitabine was effective well tolerated by all 20 participants [85]. Phase II of a similar project reported its cancer inhibitory activity and well tolerance with no side effects in patients despite its low absorption and bioavailability [86]. Efforts were made to enhance its bioavailability by either co administering drugs that suppressed intestinal and hepatic glucuronidation or by reconstituting it with other turmeric components, such as non-curcuminoids [87, 88].

A random double-blind study was conducted to evaluate the effects of curcumin on prostate-specific antigen (PSA). Eighty-five patients participated in the study. Patients were administered 100 mg of curcumin and 40 mg of isoflavones for six months. The study outcome showed that combinatory treatment reduced PSA and attenuated the activity of the androgen receptor [89]. Another double-blind study on eighty two participants reported its influence on PSA and highlighted its null side effects and well tolerability [90]. However, due to its poor bioavailability and high hydrophobicity, its use has faced huge setbacks at the clinical level. So, current efforts are aimed to enhance its bioavailability by the application of nanotechnology.

### Curcumin nanoformulations and their implications in PC

Several curcumin nanoformulations have been developed, and others are still under clinical trials assessment [91]. With the ultimate intent of enhancing the curcumin bioavailability, solubility, and absorption, such formulations have been targeted with several modifications [92]. In addition to this, several curcumin nanoformulations have been prepared to provide shielding to curcumin from hydrolysis and to increase its retention period in the body [93]. There exist few curcumin-based nanoformulations with more significant pharmaceutical potential in diagnosing various human diseases [94], a number of studies have shed light on their anti-proliferative potential [95, 96], but only a scarce amount of them have searched for the therapeutic effects of curcumin nanoformulations in PC [97]. So, here, we have summarized the curcumin nanoformulations and their significance in regulating PC cell growth.

Liposomes are small spherical vesicles consisting of phospholipid bilayers [98]. They have been extensively used as potent drug delivery systems for various biologically active substances due to their low cytotoxicity, high solubility, excessive biocompatibility, and limited biodegradability [99]. Liposome nanoformulations are easy to prepare. The size of these nanoformulations ranges from 25 nm to 2.5 mm, suggesting that they can carry almost all kinds of cargos to the target site with controlled distribution and flexibility [100]. Owing to these features, liposome-mediated nanoformulations can be considered as an efficient drug carrier for curcumin. Several studies have shed light on the fact that liposomes can incorporate curcumin in their phospholipid bilayer and ultimately increasing the curcumin distribution over the aqueous phase and its potency[93, 101]. For example, a study has demonstrated that curcumin-based liposomes could reduce PC cell progression in PC-3 human cells. The curcumin nanoencapsulation was able to hamper the survival rate of PC-3 cells in a time-dependent manner compared to free curcumin.

Moreover, liposome carriers increased the curcumin absorption in such cells, as illustrated by cell fluorescence intensity compared to controls [102]. A nanoformulation of cyclodextrin, commonly known as β-cylodextrin-curcumin (β-CD-Cur), has been found to increase the curcumin bioavailability in PC. β-CD-Cur also raised curcumin bioavailability by several folds, suggesting that such nanoformulation could be used as a potential carrier for improving curcumin delivery [103].

Surfactant free curcumin nanosphere has also been implicated in reducing cell growth in different types of cancer[104]. For example, poly D, L-lactic-co-glycolic acid (PLGA)-encapsulated curcumin nanospheres showed anti-PC activity. Cell viability assays confirmed that nanospheres of PLGA-encapsulated curcumin reduced tumor growth as compared to normal administration of curcumin. PLGA nanoparticles loaded with curcumin successfully inhibited AR and β-catenin activity in growth obstructed PC cells. Also, these nanoparticles increased apoptosis and lysosomal activity [105]. A study has recently demonstrated that curcumin-based gold nanoparticles (cur-AuNPs) could inhibit PC cell growth. However, their stability was hampered by a number of factors, including serum proteins. For instance, the addition of fetal bovine serum (FBS) to the *in vitro* PC cell models increased the curcumin bioavailability and significantly reduced the growth of PC cell lines[106]. Taken together, such findings indicate that cur-AuNPs can be used as a sustainable drug delivery platform for PC treatment. Moreover, and considering that PC-targeted curcumin nanoformulations have a tremendous ability to enhance the curcumin biocompatibility, bioavailability, specificity, and metabolism, further investigations are needed to explore these nanocarrier systems into clinical trials for PC therapy.

## Discussion

PC is a serious anomaly with dire consequences, and it has been estimated that the death toll concerning PC will rise in the coming years [1]. The currently available therapeutic options for this complicated disease are still limited and pose several side effects, which can even lead to life-threatening situations. The AR signaling has been a target of immense attention because of its involvement in PC. Indeed, the AR signaling pathway’s molecular level defects can trigger PC growth, differentiation, metastasis, invasion, and aggressiveness [107]. New insights given to miRNAs and their interactions with different signaling cascades have become more and more significant, as they are chief modulators of almost all cells’ machinery [108]. Recent literature has shed light on the fact that these micro managers could have broader implications in AR receptor signaling and can be used as feasible diagnostic or therapeutic options for PC treatment [109]. AR signaling abrogation has been responsible for tumor growth and metastasis. Several studies have delineated the role of miRNAs in AR signaling regulation at various points so that miRNAs can be targeted to restore the normal AR signaling [39, 109]. In addition, serum miRNAs levels can also be used as possible diagnostic and prognostic markers for early detection and recurrence of disease [39]. Tumor heterogeneity is a major obstacle in devising new therapeutics for PC; thus, miRNAs can be a handful in providing necessary data for culminating disease at its early stages. On the other side, miRNAs interaction with natural compounds, most specifically with curcumin, can also be explored as a new regime for potential theragnostic approaches. Curcumin has great potential to facilitate anti-proliferation and reduce PC stem cell migration by modulating miRNAs [110]. In such a way, locked nucleic acid (LNAs) approaches can prove beneficial against those miRNAs, which are overexpressed in PC and promote proliferation [111]. Stem cell modulation can also be a fruitful approach in preventing disease recurrence, despite the clear need for concrete evidence and more in-depth studies [112]. Moreover, the establishment of miRNAs as a possible therapeutic solution for PC demands extensive ground breaking studies.

Natural compounds have been in the broad attention over the decades for their potential therapeutic benefits. To date, a number of natural compounds have been under clinical trials for their effective culmination of different diseases. Various plant derivatives, such as alkaloids, flavonoids, and terpenoids, have been extensively addressed for their anti-proliferative effects on multiple cancer types [113]. Among them, a key focus has been given to curcumin for its anti-cancer, anti-inflammatory, antibacterial, and antioxidant abilities. To date, several curcumin derivatives have been under clinical trials to establish them as potential therapeutic options for various diseases [114–116]. However, little work is done on curcumin for PC treatment. One major drawback of curcumin is its limited water solubility, making it really challenging as it reduces its target-oriented efficacy and absorption [116]. In such a way, the curcumin incorporation into various nanoformulations has been viewed as a key strategy to overcome such limitations. There has been great progress on curcumin nanoformulations over the past decade. Several curcumin nanoformulations have been used to treat a plethora of human diseases [105]. The amalgamation of curcumin with suitable nano-carrier has greatly enhanced its pharmacokinetics [117], despite many questions still exist regarding drug targeted nanoformulations for PC. Current researches have devised strategies for the successful incorporation of curcumin into various nano-carriers to ameliorate its bioavailability, cell uptake, specificity, and effectiveness, but limited work has been done regarding its anti-PC activity, with most nanoformulations have not cleared the pre-clinical stages [118, 119].

Thus, as most of these nanoformulations are under pre-clinical stages, the effects of curcumin-based nanoformulations on humans are still a question that requires plenty of pondering. The limited data available from clinical trials underline the huge number of gaps in the safety and efficacy of curcumin nanoformulations for therapeutic purposes. A major concern associated with the use of nanoparticles or nanoformulation is the allergic reactions and immunogenicity. The use of curcumin alone is safe and has shown no side effects in any clinical study. However, its nanoformulation might incite immune allergic reactions. A study reported that such reactions are because of the properties of the nanoformulation used [120]. Along with robust clinical trials required to bring curcumin from bench to bedside, further investigations are necessary to evaluate the safety levels of its derivatives and nanoformulations. Finally, targeting AR signaling with miRNAs and curcumin, despite seems a promising approach, should be validated with plenty of more detailed data so that curcumin- and miRNAs-based drugs can go through clinical trials.

## Conclusions

Androgen signaling has a significant role in the progression of PC. Several studies have suggested modulating this signaling cascade through miRNAs and natural compounds such as curcumin. Few studies have delineated the curcumin potential in regulating androgen signaling in PC; however, poor bioavailability of curcumin has restricted its clinical use. Nanoformulations of curcumin have been tested in PC and other diseases for their potential. Experimental data suggests its efficacy and capability to cure PC, but rising concern for the safety and associated adverse effects of nanoformulations has presented another setback for curcumin clinical use. More *in vitro* and animal model-based studies will enhance our comprehension of the safe clinical use of curcumin. Likewise, studies targeted to reduce the toxicity of nanoparticles should be encouraged. Further, the potential of different curcumin derivatives can also be tested for their influence on the viability of PC cells.

## Data Availability

Yes.
